# Two new species of the genus *Milnesium* Doyère, 1840 (Tardigrada, Apochela, Milnesiidae) from Madagascar

**DOI:** 10.3897/zookeys.884.29469

**Published:** 2019-10-30

**Authors:** Łukasz Kaczmarek, Daria Grobys, Adam Kulpa, Tomasz Bartylak, Hanna Kmita, Marta Kepel, Andrzej Kepel, Milena Roszkowska

**Affiliations:** 1 Department of Animal Taxonomy and Ecology, Faculty of Biology, Adam Mickiewicz University, Poznań, Uniwersytetu Poznańskiego 6, 61-614 Poznań, Poland; 2 Department of Bioenergetics, Faculty of Biology, Adam Mickiewicz University, Poznań, Uniwersytetu Poznańskiego 6, 61-614 Poznań, Poland; 3 Polish Society for Nature Conservation “Salamandra”, Stolarska 7/3, 60-788 Poznań, Poland

**Keywords:** integrative taxonomy, Milnesiidae, *Milnesium matheusi* sp. nov., *Milnesium wrightae* sp. nov., tropical region

## Abstract

The knowledge of the diversity and distribution of tardigrades on Madagascar is rather poor. To date, only 13 tardigrade taxa have been reported from this region (including one *Milnesium* species). We examined 46 specimens belonging to two new-to-science species of the genus *Milnesium* described herein using an integrative approach, including classical morphology and molecular marker (COI, ITS-2 and 28S rRNA) analysis. The species were found in two moss and lichen samples collected in the Ivohibory forest in Fianarantsoa Province. *Milnesium matheusi***sp. nov.**, with claw configuration [3-3]–[3-3] and rather wide buccal tube, morphologically is most similar to: *Mil. beatae* Roszkowska, Ostrowska & Kaczmarek, 2015, *Mil. bohleberi* Bartels, Nelson, Kaczmarek & Michalczyk, 2014, *Mil. eurystomum* Maucci, 1991, *Mil. shilohae* Meyer, 2015 and *Mil. tumanovi* Pilato, Sabella & Lisi, 2016; however, it differs from these by morphometric characteristics. *Milnesium wrightae***sp. nov.**, by the presence of four points on secondary branches of claws IV, is most similar to *Mil. quadrifidum* Nederström, 1919. However, *Mil. wrightae***sp. nov.** differs from *Mil. quadrifidum* by claw configuration ([4-4]–[4-4] in *Mil. quadrifidum* vs. [3-3]–[4-4] in *Mil. wrightae***sp. nov.**), but also by the position of the fourth points on secondary branches of claws IV, which are located near the base of the claw in the new species and near the top of the claw in *Mil. quadrifidum*. Genotypic analysis showed that *Mil. matheusi***sp. nov.** is most similar to *Milnesium* sp. (28S rRNA), *Mil. variefidum* (COI) and *Mil. t. tardigradum* (ITS-2) while *Mil. wrightae***sp. nov.** is most similar to *Milnesium* sp. (28S rRNA), *Mil. variefidum* (COI) and *Mil. matheusi* (ITS-2). Five *Milnesium* taxa are recorded from the African region, including the two new species from Madagascar reported in this study.

## Introduction

Madagascar stretches from ~12° to ~26°S latitude on the Indian Ocean, more than 400 km east of Africa. With an area of ca. 590,000 km^2^, Madagascar is the world’s fourth largest island; however, it is sometimes considered a microcontinent due to its geological and biological history. First, it separated from Gondwana as part of East Gondwana, comprising the Antarctic, Madagascar, Indian, and Australian plates. After several subsequent breakups, it finally separated from the Seychelles and India ca. 66–90 My ago (de Witt 2003, Kusky et al. 2007). Madagascar is characterised by high biological endemism, estimated at >90% for terrestrial vertebrates and >80% for vascular plants (Goodman and Benstead 2003, Callmander et al. 2011). A great number of species occurring in Madagascar have restricted geographical ranges and are reported from only one or several localities (Wilmé et al. 2006). Several present taxa are assumed to be Gondwanan relicts. Most of the biota is believed to be derived from African and Asian colonizers (Yoder and Nowak 2006, Warren et al. 2010, Buerki et al. 2013; Hong-Wa and Besnard 2013). Madagascar has a tropical climate with two main climatic and biogeographic zones characterised by a substantially different vegetation cover, i.e., evergreen humid forests and deciduous forests divided by a mountain range that extends from north to south in eastern-central Madagascar (Du Puy and Moat 1996). Both zones are divided into several regions, each of which has distinctive climatic features and a set of unique habitats.

The area studied is located in south-central Madagascar (approximately 22.598830S, 46.720841E Ivohibe District, Fianarantsoa Province) on the eastern slopes of a hill located on the dry side of the main mountain range. The Ivohibory forest – which is a humid rainforest with some patches of grassy clearings – covers an area of approximately 1400 ha with an elevation gradient stretching from 900 to 1500 m asl, surrounded by human-created savannah, with a few lasting micro-patches of dry forest. It is situated on quartzite deposits, which is unique for this region (Wright and Houlihan 2017). This unusual geology strongly influences the species composition of the existing vegetation (Du Puy and Moat 1996).

The phylum Tardigrada currently consists of ca. 1200 species (Guidetti and Bertolani 2005; Degma and Guidetti 2007; Degma et al. 2009–2018; Vicente and Bertolani 2013) that inhabit terrestrial and aquatic (freshwater and marine) environments throughout the world (Ramazzotti and Maucci 1983; Nelson et al. 2015). Our knowledge of the diversity and distribution of terrestrial tardigrades on Madagascar is very poor. To date, only 13 species (*Bryodelphax parvulus* Thulin, 1928, Calcarobiotus (Discrepunguis) polygonatus (Binda & Guglielmino, 1991), *Cornechiniscus madagascariensis* Maucci, 1993, *Doryphoribius flavus* (Iharos, 1966), *Echiniscus perarmatus* Murray, 1907a, *Ech. walteri* Pilato & Lisi, 2003, *Macrobiotus hufelandi hufelandi* C.A.S. Schultze, 1834, *Mac. madegassus* Maucci, 1993, *Mesobiotus harmsworthi harmsworthi* (Murray, 1907b), *Mil. tardigradum tardigradum* Doyère, 1840, *Minibiotus intermedius* (Plate, 1888), Paramacrobiotus (Paramacrobiotus) richtersi (Murray, 1911) and *Pseudechiniscus suillus* (Ehrenberg, 1853)) have been reported from this region (Maucci 1993; Pilato and Lisi 2003).

Species of the genus *Milnesium* Doyère, 1840 are large and carnivorous, feeding mainly on rotifers, nematodes and other tardigrades, but single reports show that they can also feed on amoebas (Miller and Williams 2012; Roszkowska et al. 2015, 2016). Species in this genus reproduce parthogenetically and/or bisexually, and are characterised by sexual dimorphism (e.g., Suzuki 2003; Ciobanu et al. 2015). Thirty-eight species of the genus *Milnesium* have been reported mostly from mosses and lichens from many localities, ranging from the Antarctic through tropical and temperate to Arctic regions. Most have been described in recent years (Degma et al. 2009–2018; Kaczmarek et al. 2014, 2015, 2016; McInnes et al. 2017). According to its unique morphology (and based on molecular data) the genus *Milnesium* is classified in the class Apotardigrada (Schuster, Nelson, Grigarick & Christenberry, 1980) (Guil et al. 2019). Until now, only three *Milnesium* species (*Mil. dornensis* Ciobanu, Roszkowska & Kaczmarek, 2015, *Mil. t. tardigradum* and *Mil. tetralamellatum* Pilato & Binda, 1991) have been reported in the so-called African region (McInnes et al. 2017), in which Madagascar is placed. This paper describes two new species from Madagascar using integrative taxonomy.

## Material and methods

### Sample processing

Two moss and lichen samples from tree and rocks were collected in the Ivohibory forest on June 4, 2017 (permits No 122/17/MEEF/SG/DGF/DSAP/SCB.Re and 150N-EV06/MG17). The samples were packed in paper envelopes, dried at a temperature of ca. 30 °C and delivered to the laboratory at the Faculty of Biology, Adam Mickiewicz University, Poznań, Poland. Tardigrades were extracted from the samples and studied following the protocol of Stec et al. (2015).

### Microscopy and imaging

Specimens for light microscopy were mounted on microscope slides in a small drop of Hoyer’s medium, prepared according to Ramazzotti and Maucci (1983) as in the English translation by Beasley (1995), and secured with a cover slip. The slides were then placed in an incubator and dried for two days at ca. 60 °C. Dried slides were sealed with a transparent nail polish and examined under an Olympus BX41 phase contrast light microscope (PCM) associated with an ARTCAM–300Mi digital camera (Olympus Corporation, Shinjuku-ku, Japan).

All figures were assembled in Corel Photo-Paint 2017. For deep structures that could not be fully focused in a single photograph, a series of 2–10 images were taken every ca. 0.5 μm and then manually assembled into a single deep-focus image in Corel Photo-Paint 2017.

### Morphometrics and morphological nomenclature

All measurements are given in micrometres [μm]. Structures were measured only if their orientation was suitable. Body length was measured from the anterior extremity to the end of the body, excluding the hind legs. All measurements (except buccal tube width) followed protocols in Tumanov (2006). Buccal tube width was measured at three points as suggested by Michalczyk et al. (2012). The *pt* ratio is the ratio of the length of a given structure to the length of the buccal tube, expressed as a percentage (Pilato 1981). The *pt* values are always provided in [square brackets and in italics]. Configuration of the number of claw points on the secondary branches (“claw configuration”) is given according to Michalczyk et al. (2012).

Morphometric data were handled using the “Apochela” ver. 1.1 template available from the Tardigrada Register (Michalczyk and Kaczmarek 2013). Tardigrade taxonomy follows Bertolani et al. (2014) and Guil et al. (2019). Genus abbreviations follow Perry et al. (2019).

### Comparative material

Species were identified using the key in Morek et al. (2016) and other original descriptions/re-descriptions (Nederström 1919; Maucci 1991; Bartels et al. 2014; Meyer 2015; Roszkowska et al. 2015; Pilato et al. 2016), or based on direct examination of fixed specimens (holotype and paratypes of *Mil. bohleberi* and specimens of *Mil. eurystomum* from Spitsbergen, Department of Animal Taxonomy and Ecology, Adam Mickiewicz University, Poznań, Poland). Tardigrade taxonomy follows Marley et al. (2011).

### Genotyping

All specimens were preliminarily identified using light microscopy (LM) before DNA extraction. Later, each specimen was placed individually in a 1.5 ml Eppendorf microcentrifuge tube in 20 µl of sterile MQ H_2_O and kept frozen at -80 °C until DNA isolation. DNA was extracted from individual animals following a modified Chelex100 resin (Bio-Rad) extraction method (Casquet et al. 2012), modified in order to obtain tardigrade exoskeletons, according to Zawierucha et al. (2016). DNA was extracted by incubating each specimen in 40 µl of 10% Chelex100 resin solution in sterile MQ H_2_O with the addition of 0.02 mg of Proteinase K (Genoplast) at 55 °C for 5h with shaking (500 RPM, Eppendorf Thermomixer 5436) and occasionally centrifuged. In the next step, Proteinase K was inactivated by incubating at 70 °C for 15 min. Subsequently, 20 µl of sterile MQ H_2_O was added to the tube and centrifuged for 2 min at 8000 G. For further analysis, ca. 40 µl of DNA extract (to the level of remaining Chelex beads at the bottom) was carefully transferred from each tube to a new 1.5 ml Eppendorf microcentrifuge tube. The tardigrade exoskeleton, present in a pellet after centrifugation, containing Chelex beads on the bottom of each tube, was extracted under stereomicroscope and then mounted in Hoyer’s medium for further morphological analysis. Polymerase chain reaction (PCR) amplifications were carried out for three DNA fragments differing in mutation rates: mitochondrial cytochrome oxidase subunit I (COI), nuclear internal transcribed spacer 2 (ITS-2) and cytoplasmic ribosome large subunit component (28S rRNA) in a total volume of 15–50 μl (see Table 1 for primers, Table 2 for PCR cocktail recipes and Table 3 for PCR programmes). PCR products were verified by agarose gel electrophoresis (1–1.2% agarose) with ethidium bromide. Prior to sequencing, PCR products were purified with thermosensitive Exonuclease I and FastAP Alkaline Phosphatase (Fermentas, Thermo Scientific) to improve their quality. Properly prepared PCR products were sequenced bidirectionally with BigDye Terminator v3.1 on an ABI Prism 3130XL Analyzer (Applied Biosystems, Foster City, CA, USA), according to the manufacturer’s protocol. The sequences were edited and manually checked against non-conservative alignments using BioEdit, version 7.0.5 (Hall 1999), and submitted to GenBank (see Results section).

**Table 1.** Primers used for amplification and sequencing of DNA fragments.

DNA fragment	Direction	Code	Sequence (5’-3’)	Reference
COI	Forvard	bcdF01	CATTTTCHACTAAYCATAARGATATTGG	Dabert et al. 2010
	Reverse	bcdR04	TATAAACYTCDGGATGNCCAAAAAA	Dabert et al. 2008
ITS-2	Forvard	ITS2_Eutar_Ff	CGTAACGTGAATTGCAGGAC	Stec et al. 2018
	Reverse	ITS2_Eutar_Rr	TGATATGCTTAAGTTCAGCGG	
28S rRNA	Forvard	28SF0001	ACCCVCYNAATTTAAGCATAT	Mironov et al. 2012
	Reverse	28SR0990	CCTTGGTCCGTGTTTCAAGAC	

**Table 2.** PCR cocktails used for the amplification of DNA fragments.

Component	Concentration	Additional note
H_2_O	–	sterile MQ
buffer	1×	5X Phusion HF Buffer; Thermo Scientific
dNTPs	200 µM	dNTP Mix; Thermo Scientific
forward primer	0.5 µM	–
reverse primer	0.5 µM	–
polymerase	0.02 U/µl	Phusion High-Fidelity DNA Polymerase; Thermo Scientific
DNA	–	–

**Table 3.** PCR programmes used for the amplification of COI, ITS-2 and 28S rRNA.

Step	COI			ITS-2 and 28S rRNA		
	Cycles	Time [min.:sec.]	Temp. [°C]	Cycles	Time [min:sec]	Temp. [°C]
initial denaturation	–	05:00	98	–	05:00	98
denaturation	5	00:30	98	–	–	–
annealing		00:30	45	–	–	–
extension		01:00	72	–	–	–
denaturation	30	00:30	98	35	00:30	98
annealing		00:30	50		00:30	50
extension		01:00	72		01:00	72
final extension	–	07:00	72	–	07:00	72

### Comparative molecular analysis

In the first step, the sequences of *Mil. wrightae* sp. nov. and *Mil. matheusi* sp. nov. were analysed by Standard Nucleotide BLAST to confirm their uniqueness. Then, a comparison was performed with COI, ITS-2 and 28S rRNA sequences of the genus *Milnesium*, deposited in GenBank, using only the sequences of good quality and length. All sequences were aligned with the ClustalW Multiple Alignment tool (Thompson et al. 1994) implemented in BioEdit and trimmed to 510 (COI), 184 (ITS-2), 625 (28S rRNA) bp, respectively. Based on the recommendation of Srivathsan and Meier (2012), pairwise distances were calculated using MEGA7 in preference to the genetic distances corrected by the Kimura 2 parametric model (K2P). All positions with less than 95% site coverage were eliminated.

## Results

### Taxonomic account

#### Phylum Tardigrada Doyère, 1840

**Class Apotardigrada (Schuster, Nelson, Grigarick & Christenberry, 1980)**

**Order Apochela Schuster, Nelson, Grigarick & Christenberry, 1980**

**Family Milnesiidae Ramazzotti, 1962**

**Genus *Milnesium* Doyère, 1840**

##### Milnesium matheusi sp. nov.

Taxon classificationAnimaliaApochelaMilnesiidae

7A1EBE3B-675A-56AD-AE2B-15B8255DCAB3

http://zoobank.org/3EB072A7-1C84-4EF6-B6D2-D9486BBF6C4F

**Figures 1–3.**
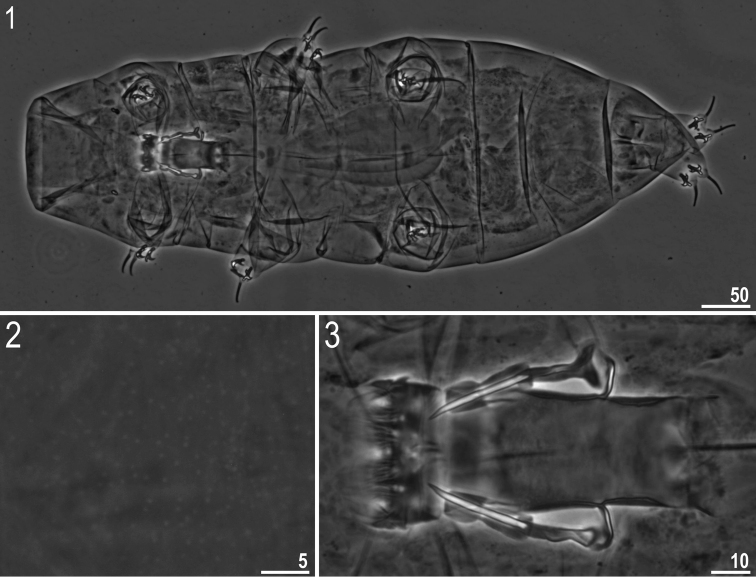
*Milnesium matheusi* sp. nov. **1** Habitus (ventral view) (holotype) **2** dorsal cuticle with pseudopores (holotype) **3** buccal tube (holotype). All in PCM.

**Figures 4, 5.**
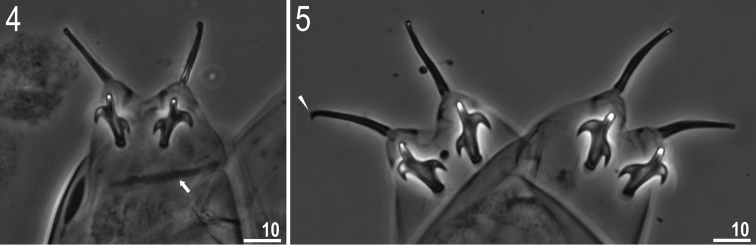
*Milnesium matheusi* sp. nov. **4** Claws II (paratype), arrow indicates bar under claw **5** claws IV (holotype), arrowhead indicates small accessory point. All in PCM.

Figures 1–3 , 4–5 , Tables 4 , 5

###### Material examined.

Holotype and 18 paratypes, all from sample No 139: Ivohibory forest, Madagascar, lichen sample from quartz rocks, coll. Marta Kepel and Andrzej Kepel.

###### Description.

***Adult females*** (Fig. 1, Table 4) with no modified claws I. Body light yellow before fixation and transparent afterwards, eyes present (in 89% of measured specimens). Dorsal cuticle sculptured with pseudopores, not arranged in bands, sparsely distributed and not forming a reticular design (Fig. 2). Six peribuccal papillae and six peribuccal lamellae present around the mouth opening. Two cephalic papillae positioned laterally. Peribuccal papillae slightly longer than lateral papillae.

The buccal apparatus of the *Milnesium* type (Figs 1, 3). The buccal tube wide and short (standard width, on average 46% of its length), and slightly funnel-shaped, wider anteriorly (posterior diameter on average 89% of the anterior diameter) (Table 4). The pharyngeal bulb elongated, pear-shaped and without placoids or septulum.

**Table 4.** Measurements and *pt* values of selected morphological structures of adult females of *Milnesium matheusi***sp. nov**. mounted in Hoyer’s medium (N – number of specimens/structures measured, RANGE refers to the smallest and the largest structure among all measured specimens; SD – standard deviation, *pt* – ratio of the length of a given structure to the length of the buccal tube expressed as a percentage).

Character	N	Range						Mean		SD		Holotype	
		µm			*pt*			µm	*pt*	µm	*pt*	µm	*pt*
Body length	6	630	–	766	–	–	–	691	–	45	–	766	–
Peribuccal papillae length	5	10.0	–	12.0	*18.6*	–	*22.1*	11.0	*19.9*	0.8	*1.5*	11.8	*18.9*
Lateral papillae length	7	9.4	–	10.7	*16.5*	–	*19.7*	10.0	*18.1*	0.4	*1.2*	10.3	*16.5*
Buccal tube													
Length	9	51.3	–	62.5	–	–	–	56.6	–	3.8	–	62.5	–
Stylet support insertion point	9	34.5	–	42.3	*66.1*	–	*69.4*	38.4	*67.8*	2.4	*1.3*	41.5	*66.4*
Anterior width	9	25.2	–	35.9	*47.6*	–	*57.9*	28.9	*51.0*	3.2	*3.1*	31.4	*50.2*
Standard width	9	23.1	–	31.1	*42.4*	–	*50.8*	26.3	*46.5*	2.7	*3.0*	29.4	*47.0*
Posterior width	9	23.0	–	30.2	*41.1*	–	*50.3*	25.7	*45.3*	2.6	*3.1*	28.9	*46.2*
Standard width/length ratio	9	42%	–	51%	–	–	–	46%	–	3%	–	47%	–
Posterior/anterior width ratio	9	84%	–	94%	–	–	–	89%	–	4%	–	92%	–
Claw 1 lengths													
External primary branch	9	17.2	–	21.8	*30.2*	–	*35.2*	18.9	*33.3*	1.5	*1.6*	21.8	*34.9*
External base + secondary branch	9	13.3	–	16.7	*23.5*	–	*27.9*	15.0	*26.5*	1.2	*1.5*	16.6	*26.6*
External spur	7	3.5	–	5.3	*6.4*	–	*9.6*	4.4	*7.8*	0.7	*1.3*	?	?
External branches length ratio	9	76%	–	82%	–	–	–	80%	–	2%	–	76%	–
Internal primary branch	9	16.0	–	21.1	*30.2*	–	*34.5*	18.3	*32.3*	1.6	*1.6*	21.1	*33.8*
Internal base + secondary branch	9	13.3	–	16.6	*24.5*	–	*27.3*	14.8	*26.2*	1.1	*1.0*	16.3	*26.1*
Internal spur	9	3.3	–	5.5	*6.1*	–	*10.5*	4.4	*7.7*	0.8	*1.4*	5.5	*8.8*
Internal branches length ratio	9	77%	–	88%	–	–	–	81%	–	4%	–	77%	–
Claw 2 lengths													
External primary branch	8	17.4	–	21.2	*32.9*	–	*36.5*	19.5	*34.9*	1.4	*1.4*	21.2	*33.9*
External base + secondary branch	7	13.7	–	17.0	*24.5*	–	*27.5*	15.0	*26.7*	1.1	*1.2*	17.0	*27.2*
External spur	3	3.9	–	4.9	*7.2*	–	*7.8*	4.4	*7.6*	0.5	*0.4*	4.9	*7.8*
External branches length ratio	7	72%	–	81%	–	–	–	77%	–	3%	–	80%	–
Internal primary branch	8	16.8	–	20.5	*31.1*	–	*35.7*	18.7	*33.3*	1.3	*1.5*	20.2	*32.3*
Internal base + secondary branch	9	13.0	–	16.3	*25.0*	–	*27.9*	14.7	*26.0*	1.1	*0.9*	16.3	*26.1*
Internal spur	9	3.4	–	5.8	*6.1*	–	*10.3*	4.4	*7.8*	0.8	*1.5*	4.7	*7.5*
Internal branches length ratio	8	74%	–	81%	–	–	–	78%	–	3%	–	81%	–
Claw 3 lengths													
External primary branch	5	19.7	–	21.0	*32.3*	–	*38.3*	20.5	*35.7*	0.6	*2.5*	?	?
External base + secondary branch	6	14.2	–	16.3	*24.5*	–	*28.4*	15.4	*27.1*	0.7	*1.5*	?	?
External spur	5	3.5	–	5.2	*6.4*	–	*9.3*	4.4	*7.7*	0.7	*1.1*	?	?
External branches length ratio	5	72%	–	82%	–	–	–	75%	–	4%	–	?	–
Internal primary branch	5	18.9	–	20.4	*31.3*	–	*36.5*	19.7	*34.3*	0.6	*2.0*	?	?
Internal base + secondary branch	6	13.7	–	16.0	*23.7*	–	*28.2*	14.9	*26.2*	0.8	*1.9*	?	?
Internal spur	5	3.8	–	5.6	*7.0*	–	*9.7*	4.8	*8.3*	0.7	*1.1*	?	?
Internal branches length ratio	5	70%	–	79%	–	–	–	75%	–	4%	–	?	–
Claw 4 lengths													
Anterior primary branch	7	19.6	–	23.0	*35.1*	–	*39.8*	20.9	*37.2*	1.3	*1.5*	23.0	*36.8*
Anterior base + secondary branch	7	14.6	–	17.2	*26.3*	–	*29.4*	15.8	*28.2*	0.9	*1.1*	17.2	*27.5*
Anterior spur	6	4.1	–	6.3	*7.5*	–	*11.5*	5.4	*9.7*	0.9	*1.6*	6.0	*9.6*
Anterior branches length ratio	7	71%	–	80%	–	–	–	76%	–	4%	–	75%	–
Posterior primary branch	7	20.5	–	24.0	*38.1*	–	*41.3*	21.8	*38.9*	1.1	*1.1*	24.0	*38.4*
Posterior base + secondary branch	7	15.2	–	17.7	*26.9*	–	*29.6*	16.1	*28.6*	0.8	*1.0*	17.7	*28.3*
Posterior spur	7	4.4	–	5.8	*7.6*	–	*10.3*	5.2	*9.3*	0.6	*1.1*	5.5	*8.8*
Posterior branches length ratio	7	70%	–	76%	–	–	–	74%	–	2%	–	74%	–

Claws of the *Milnesium* type, slender (Figs 4, 5). Primary branches on all legs with small, but distinct accessory points detaching from the branch at its greatest curvature (Fig. 5, arrowhead). Secondary branches with rounded basal thickenings (Figs 4, 5). All secondary branches on all legs with three points (claw configuration: [3-3]–[3-3]). Single, long transverse, cuticular bars present under claws I–III (Fig. 4, arrow).

***Adult males*** (Table 5) with modified claws I. Similar to females but clearly smaller, with secondary branches of claws I modified into strong hooks and with a different proportion of peribuccal and lateral papillae length (peribuccal papillae clearly shorter than lateral), eyes present only in 33% of measured specimens.

***Eggs*** oval, smooth and deposited in the exuvium as in all other known *Milnesium* species.

**Table 5.** Measurements and *pt* values of selected morphological structures of adult males (with modified claws I) of *Milnesium matheusi* sp. nov. mounted in Hoyer’s medium (N – number of specimens/structures measured, RANGE refers to the smallest and the largest structure among all measured specimens; SD – standard deviation, *pt* – ratio of the length of a given structure to the length of the buccal tube expressed as a percentage).

Character	N	Range						Mean		SD	
		µm			*pt*			µm	*pt*	µm	*pt*
Body length	2	409	–	428	–	–	–	419	–	13	–
Peribuccal papillae length	3	3.0	–	3.9	*8.9*	–	*11.3*	3.5	*10.2*	0.5	*1.2*
Lateral papillae length	3	5.6	–	6.0	*16.2*	–	*17.8*	5.9	*17.1*	0.2	*0.8*
Buccal tube											
Length	3	33.8	–	34.5	–	–	–	34.2	–	0.4	–
Stylet support insertion point	2	21.2	–	22.3	*62.7*	–	*64.6*	21.8	*63.7*	0.8	*1.4*
Anterior width	3	9.4	–	11.2	*27.8*	–	*32.6*	10.5	*30.8*	1.0	*2.6*
Standard width	3	9.1	–	9.8	*26.9*	–	*28.5*	9.5	*27.8*	0.4	*0.8*
Posterior width	3	9.4	–	10.2	*27.8*	–	*29.6*	9.8	*28.7*	0.4	*0.9*
Standard width/length ratio	3	27%	–	28%	–	–	–	28%	–	1%	–
Posterior/anterior width ratio	3	88%	–	100%	–	–	–	94%	–	6%	–
Claw 1 lengths											
External primary branch	2	15.8	–	16.3	*45.9*	–	*48.2*	16.1	*47.1*	0.4	*1.6*
External base + secondary branch	3	14.1	–	15.0	*41.7*	–	*43.5*	14.6	*42.7*	0.5	*0.9*
External spur	2	3.2	–	3.4	*9.3*	–	*9.9*	3.3	*9.6*	0.1	*0.4*
External branches length ratio	2	87%	–	94%	–	–	–	90%	–	5%	–
Internal primary branch	3	14.9	–	15.7	*43.2*	–	*46.4*	15.4	*45.1*	0.5	*1.7*
Internal base + secondary branch	3	14.0	–	14.5	*40.6*	–	*42.9*	14.2	*41.4*	0.3	*1.3*
Internal spur	3	3.0	–	3.7	*8.9*	–	*10.7*	3.4	*9.9*	0.4	*0.9*
Internal branches length ratio	3	89%	–	94%	–	–	–	92%	–	2%	–
Claw 2 lengths											
External primary branch	2	16.9	–	17.9	*49.0*	–	*53.0*	17.4	*51.0*	0.7	*2.8*
External base + secondary branch	1	13.2	–	13.2	*39.1*	–	*39.1*	13.2	*39.1*	?	?
External spur	1	3.5	–	3.5	*10.4*	–	*10.4*	3.5	*10.4*	?	?
External branches length ratio	1	74%	–	74%	–	–	–	74%	–	?	–
Internal primary branch	3	16.4	–	16.9	*47.5*	–	*50.0*	16.7	*48.8*	0.3	*1.2*
Internal base + secondary branch	2	12.7	–	12.8	*37.2*	–	*37.6*	12.8	*37.4*	0.1	*0.3*
Internal spur	2	3.5	–	5.0	*10.4*	–	*14.5*	4.3	*12.4*	1.1	*3.0*
Internal branches length ratio	2	75%	–	76%	–	–	–	76%	–	1%	–
Claw 3 lengths											
External primary branch	3	16.2	–	17.4	*47.1*	–	*51.5*	16.8	*49.0*	0.6	*2.3*
External base + secondary branch	2	12.1	–	12.8	*35.2*	–	*37.9*	12.5	*36.5*	0.5	*1.9*
External spur	1	3.9	–	3.9	*11.3*	–	*11.3*	3.9	*11.3*	?	?
External branches length ratio	2	74%	–	75%	–	–	–	74%	–	1%	–
Internal primary branch	3	14.8	–	17.0	*43.0*	–	*50.3*	16.0	*46.7*	1.1	*3.6*
Internal base + secondary branch	2	12.7	–	13.0	*37.6*	–	*37.8*	12.9	*37.7*	0.2	*0.2*
Internal spur	2	2.9	–	4.0	*8.4*	–	*11.8*	3.5	*10.1*	0.8	*2.4*
Internal branches length ratio	2	75%	–	88%	–	–	–	81%	–	9%	–
Claw 4 lengths											
Anterior primary branch	3	16.3	–	17.0	*47.4*	–	*49.3*	16.6	*48.5*	0.4	*1.0*
Anterior base + secondary branch	2	12.4	–	12.9	*36.7*	–	*37.5*	12.7	*37.1*	0.4	*0.6*
Anterior spur	1	3.8	–	3.8	*11.0*	–	*11.0*	3.8	*11.0*	?	?
Anterior branches length ratio	2	75%	–	79%	–	–	–	77%	–	3%	–
Posterior primary branch	3	17.7	–	18.8	*51.3*	–	*54.7*	18.3	*53.4*	0.6	*1.8*
Posterior base + secondary branch	3	12.7	–	13.7	*37.1*	–	*39.8*	13.1	*38.2*	0.6	*1.5*
Posterior spur	2	3.0	–	4.1	*8.7*	–	*11.9*	3.6	*10.3*	0.8	*2.3*
Posterior branches length ratio	3	69%	–	73%	–	–	–	72%	–	2%	–

###### DNA sequences.

We obtained good quality sequences for the applied molecular markers: 28S rRNA sequence (GenBank: MN191503), 756 bp long; COI sequence (GenBank: MN187056), 628 bp long; ITS-2 sequence (GenBank: MN239906), 218 bp long.

###### Type locality.

Madagascar, 22°37'07.7"S, 46°43'14.5"E, ca. 1187 m asl, Fianarantsoa Province, Ivohibory forest.

###### Etymology.

The second author with great pleasure dedicates this species to her fiance – Mateusz Wojciechowski.

###### Type depositories.

The holotype and 13 paratypes (slides: MAD139/14, MAD139/16, MAD139/18, MAD139/19, MAD139/34, MAD139/35, MAD139/42, MAD139/56, MAD139/72) are deposited at the Department of Animal Taxonomy and Ecology, Adam Mickiewicz University in Poznań, Uniwersytetu Poznańskiego 6, Poznań, Poland; five paratypes (slides: MAD139/12, MAD139/13, MAD139/15) are deposited at the Natural History Museum, University of Copenhagen, Universitetsparken 15, DK-2100 Copenhagen, Denmark.

###### Morphological differential diagnosis.

The new species with three points on the secondary branches of all claws (claw configuration [3-3]–[3-3]) and a rather wide buccal tube, in relation to its length, is most similar to: *Mil. beatae* Roszkowska, Ostrowska & Kaczmarek, 2015, *Mil. bohleberi* Bartels, Nelson, Kaczmarek & Michalczyk, 2014, *Mil. eurystomum* Maucci, 1991, *Mil. shilohae* Meyer, 2015 and *Mil. tumanovi* Pilato, Sabella & Lisi, 2016, but it differs from:

1. *Milnesium beatae*, only reported from Argentina and USA (Roszkowska et al. 2015; Tibbs et al. 2016) by: narrower buccal tube (25.2–35.9 [*47.6–57.9*] and 23.1–31.1 [*42.4–50.8*] anterior and standard width, respectively, in the new species vs. 37.0–53.5 [*70.3–78.9*] and 32.0–42.5 [*58.1–65.6*] anterior and standard width respectively in *Mil. beatae*), smaller standard width/length ratio of the buccal tube (42%–51% in new species *vs.* 58%–66% in *Mil. beatae*) and larger posterior/anterior width ratio (84%–94% in new species vs. 69%–76% in *Mil. beatae*).

2. *Milnesium bohleberi*, only recorded from North Carolina and Tennessee (USA) (Bartels et al. 2014) by: presence of pseudopores on dorsal cuticle, shorter peribuccal papillae (10.0–12.0 [*18.6–22.1*] in new species *vs.* 15.5–20.3 [*27.2–32.3*] in *Mil. bohleberi*), smaller *pt* values of anterior, standard and posterior widths of the buccal tube (*47.6–57.9*, *42.4–50.8*, *41.1–50.3*, respectively, in new species vs. *63.4–74.7*, *54.5–64.0*, *52.4–62.0*, respectively, in *Mil. bohleberi*), smaller standard width/length ratio of the buccal tube (42%–51% in new species vs. 54%–64% in *Mil. bohleberi*) and slightly shorter claws (see Table 4 below and Bartels et al. (2014: Table 1) for the exact differences in claw dimensions).

3. *Milnesium eurystomum* reported from a few localities in Argentina, Chile, Greenland, Mongolia and USA (see review by Kaczmarek et al. 2016) by: shorter buccal tube (51.3–62.5 in new species *vs.* 70.8–77.5 in *Mil. eurystomum*), stylet supports inserted in a more posterior position (*pt = 66.1–69.4* in new species vs. ca. *pt = 60.0–60.3* in *Mil. eurystomum*), narrower buccal tube (25.2–35.9 [*47.6–57.9*], 23.1–31.1 [*42.4–50.8*] and 23.0–30.2 [*41.1–50.3*] anterior, standard and posterior width, respectively, in new species *vs.* 53.7–55.9 [*72.1–75.8*], 45.9–47.9 [*61.8–64.8*] and 33.9–41.0 [*43.7–57.9*] anterior, standard and posterior width, respectively, in *Mil. eurystomum*), smaller standard width/length ratio of the buccal tube (42%–51% in new species vs. 62%–65% in *Mil. eurystomum*) and larger posterior/anterior width ratio (84%–94% in new species vs. 61%–76% in *Mil. eurystomum*).

4. *Milnesium shilohae*, only reported from the type locality in Hawaii (USA) (Meyer 2015) by: presence of pseudopores on dorsal cuticle, presence of similar in length spurs on internal and external claws (internal and posterior spurs larger than external and anterior spurs in *Mil. shilohae*), slightly longer lateral papillae (9.4–10.7 in new species *vs.* 5.0–9.0 in *Mil. shilohae*), slightly longer buccal tube (51.3–62.5 in new species vs. 38.4–50.3 in *Mil. shilohae*), stylet supports inserted in a more anterior position (*pt = 66.1–69.4* in new species vs. *pt = 75.5–77.5* in *Mil. shilohae*) and larger spurs on some external and anterior claws (see Table 4 below and Table 3 in Meyer (2015) for the exact differences in claw dimensions).

5. *Milnesium tumanovi*, only recorded from the type locality in the Crimea (Ukraine) (Pilato et al. 2016) by: presence of pseudopores on dorsal cuticle, funnel-shaped buccal tube (cylindrical in *Mil. tumanovi*) and stylet supports inserted in a more posterior position (*pt = 66.1–69.4* in new species vs. ca. *pt = 52–54* in *Mil. tumanovi*).

###### Genotypic differential diagnosis.

The ranges of uncorrected genetic p-distances between *Mil. matheusi* sp. nov. and species of the genus *Milnesium*, for which molecular marker sequences are available from GenBank (see Table 6 for details), are as follows:

**Table 6.** Sequences of 28S rRNA, COI and ITS-2 of *Milnesium* taxa available in GenBank and used in differential diagnosis.

DNA marker	Taxon	Accession number	Source
28S rRNA	*Milnesium* sp.	JX888585.1	Adams et. al. unpublished
		JX888586.1	Adams et. al. unpublished
		JX888587.1	Adams et. al. unpublished
	*Milnesium tardigradum*	JX888541.1	Adams et. al. unpublished
		JX888540.1	Adams et. al. unpublished
		KC138808.1	Zawierucha unpublished
		KC138809.1	Zawierucha unpublished
	*Milnesium* sp.	AY210826.1	Mallatt et. al. 2004
	*Milnesium tardigradum*	FJ435780.1	Guil and Giribet 2012
		FJ435779.1	Guil and Giribet 2012
	*Milnesium berladnicorum*	KT951661.1	Morek et. al. 2016
	*Milnesium variefidum*	KT951665.1	Morek et. al. 2016
COI	*Milnesium* sp.	KX306950.1	Fox et al. unpublished
	*Milnesium tardigradum*	EU244603.1	Schill unpublished
		EU244604	Schill unpublished
		FJ435810.1	Guil and Giribet 2012
	*Milnesium t. tardigradum*	JN664950.1	Michalczyk et al. 2012
	Milnesium cf. tardigradum	JX683824.1	Vicente et al. 2013
		JX683823.1	Vicente et al. 2013
		JX683822.1	Vicente et al. 2013
	*Milnesium* sp.	KJ857002.1	Velasco-Castrillón et al. 2015
		KJ857001.1	Velasco-Castrillón et al. 2015
	Milnesium cf. alpigenum	KU513422.1	Kosztyła et al. 2016
	*Milnesium variefidum*	KT951663.1	Morek et al. 2016
	*Milnesium berladnicorum*	KT951659.1	Morek et al. 2016
	*Milnesium* sp.	EF632553.1	Sands et. al unpublished
	Milnesium cf. granulatum	MH751517.1	Jackson and Meyer 2019
	*Milnesium lagniappe*	MH751518.1	Jackson and Meyer 2019
	*Milnesium tardigradum*	MG923558.1	Morek et al. 2019
		MG923559.1	Morek et al. 2019
		MG923560.1	Morek et al. 2019
		MG923561.1	Morek et al. 2019
		MG923562.1	Morek et al. 2019
		MG923563.1	Morek et al. 2019
		MG923564.1	Morek et al. 2019
		MG923565.1	Morek et al. 2019
	*Milnesium dornensis*	MG923566.1	Morek et al. 2019
ITS-2	*Milnesium alpigenum*	MH000382.1	Morek et al. unpublished
	*Milnesium* sp.	MH000386.1	Morek et al. unpublished
		MH000387.1	Morek et al. unpublished
	*Milnesium tardigradum*	HM150648.2	Wełnicz et. al. 2010
		GQ403682.1	Schill et al. 2010
		GQ403681.1	Schill et al. 2010
	*Milnesium t. tardigradum*	JF951049	Michalczyk et al. 2012
	*Milnesium variefidum*	KT951667.1	Morek et. al. 2016
		KT951666.1	Morek et. al. 2016
	*Milnesium berladnicorum*	KT951662.1	Morek et. al. 2016
	Milnesium cf. granulatum	MK681875.1	Jackson and Meyer 2019
		MK681876.1	Jackson and Meyer 2019
		MK681877.1	Jackson and Meyer 2019
		MK681878.1	Jackson and Meyer 2019
		MK681879.1	Jackson and Meyer 2019
		MK681880.1	Jackson and Meyer 2019
		MK681881.1	Jackson and Meyer 2019
		MK681882.1	Jackson and Meyer 2019
		MK681883.1	Jackson and Meyer 2019
		MK681884.1	Jackson and Meyer 2019
		MK681885.1	Jackson and Meyer 2019
		MK681886.1	Jackson and Meyer 2019

1. 28S rRNA: 4.5–6.7% (5.4% on average), with the most similar being *Milnesium* sp. from North America (JX888585.1, JX888586.1, JX888587.1) (unpublished) and the least similar being *Mil. wrightae* sp. nov. (MN191504.1) (present studies);

2. COI: 20.1–38.8% (23.3% on average), with the most similar being *Mil. variefidum* Morek, Gąsiorek, Stec, Blagden & Michalczyk, 2016 from UK (KT951663.1) (Morek et al. 2016) and the least similar being *Mil. t. tardigradum* from Spain (FJ435810.1) (Guil and Giribet 2012);

3. ITS-2: 17.8–31.1% (23.7% on average), with the most similar being *Mil. t. tardigradum* from Germany (JF951049.1) (Michalczyk et al. 2012) and the least similar being Mil. cf. granulatum from USA (MK681879.1) (Jackson and Meyer 2019).

##### Milnesium wrightae sp. nov.

Taxon classificationAnimaliaApochelaMilnesiidae

6C85FDEA-1AF3-5410-A1BD-DCCE13323850

http://zoobank.org/A62CF1FF-8BDA-42D1-A090-6AE72608E529

**Figures 6–8.**
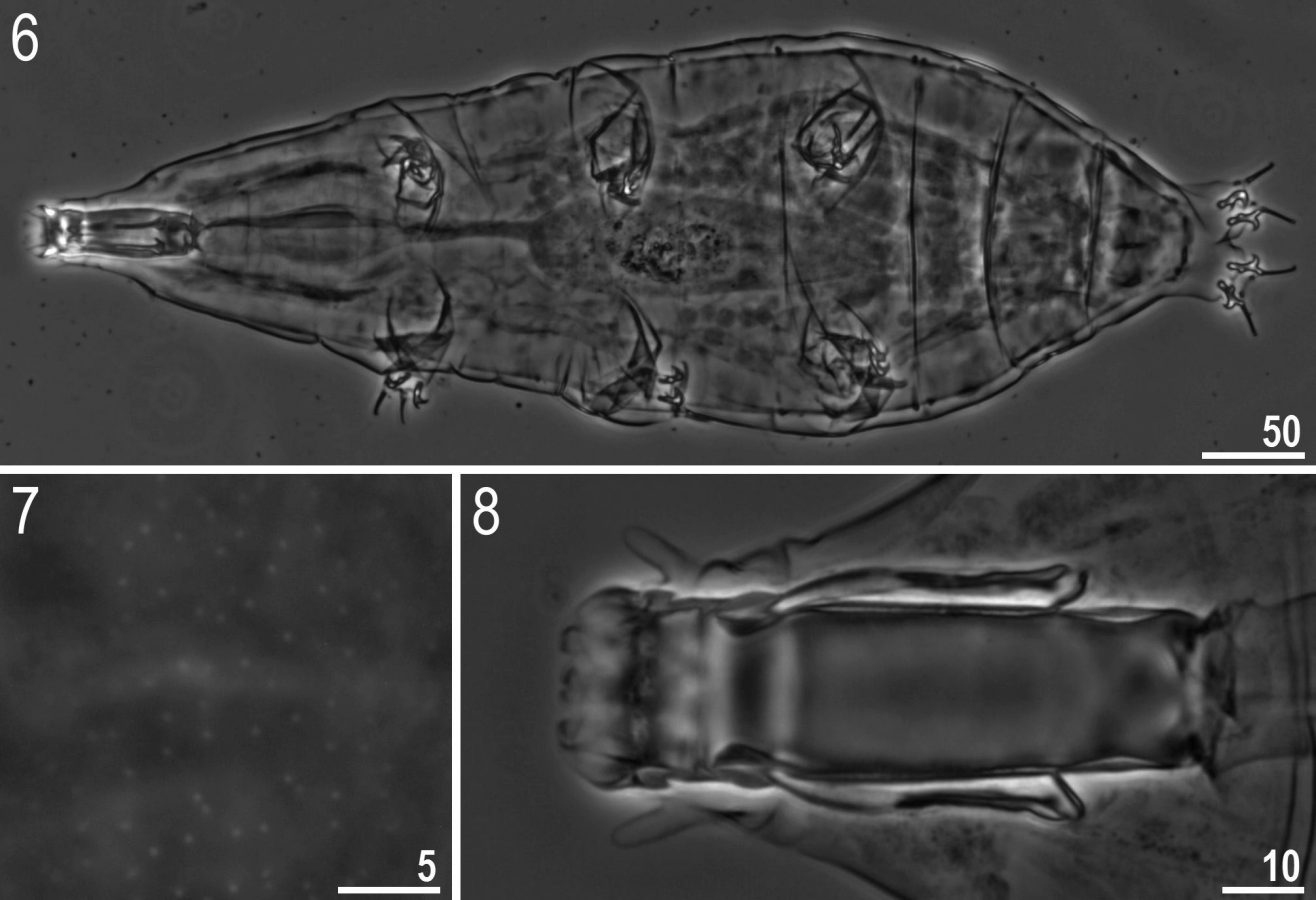
*Milnesium wrightae* sp. nov. **6** Habitus (ventral view) (holotype) **7** dorsal cuticle with pseudopores (holotype) **8** buccal tube (paratype). All in PCM.

**Figures 9–11.**
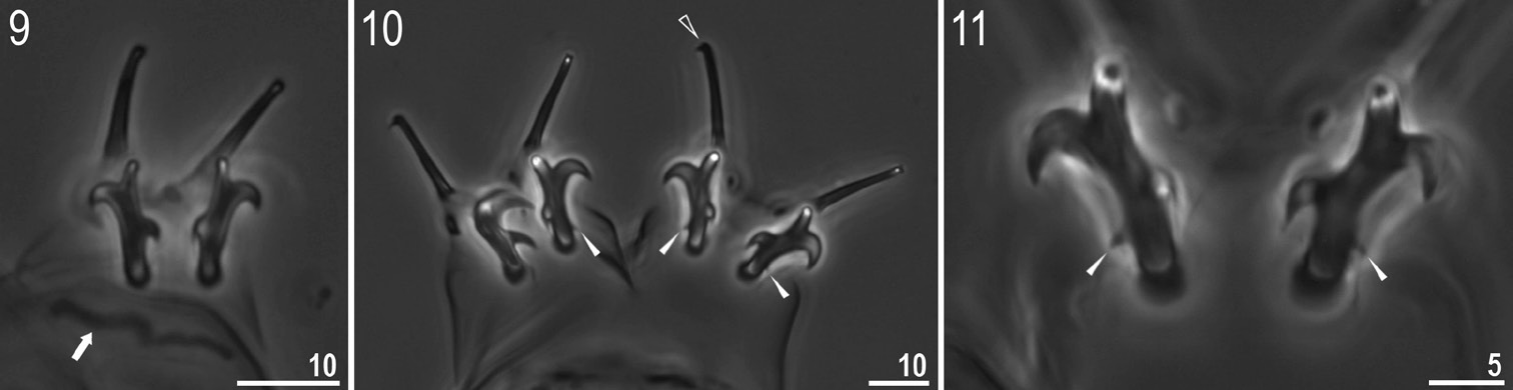
*Milnesium wrightae* sp. nov. **9** Claws I (paratype), arrow indicates bar under claw **10** claws IV (holotype), empty arrowhead indicates small accessory point, filled arrowheads indicate the fourth points on secondary branches near the base of the claw **11** focus on the fourth points on secondary branches near the base of the claw IV (holotype, filled arrowheads). All in PCM.

Figures 6–8 , 9–11 , Table 7

###### Material examined.

Holotype and 28 paratypes, all from sample No 109: Ivohibory forest, Madagascar, moss sample from tree, coll. Marta Kepel and Andrzej Kepel.

###### Description.

***Adult females*** (Fig. 6, Table 7) with no modified claws I. Body light yellow before fixation and transparent afterwards, eyes present only in 39% of measured specimens. Dorsal cuticle sculptured with pseudopores not arranged in bands, sparsely distributed and not forming reticular design (Fig. 7). Six peribuccal papillae and six peribuccal lamellae present around the mouth opening. Two cephalic papillae positioned laterally. Peribuccal papillae slightly longer than lateral papillae.

The buccal apparatus of the *Milnesium* type (Figs 6, 8). The buccal tube with standard width on average 62% of its length, and funnel-shaped, wider anteriorly (posterior diameter on average 91% of the anterior diameter) (Table 7). The pharyngeal bulb elongated, pear-shaped and without placoids or septulum.

**Table 7.** Measurements and *pt* values of selected morphological structures of females of *Milnesium wrightae* sp. nov. mounted in Hoyer’s medium (N – number of specimens/structures measured, RANGE refers to the smallest and the largest structure among all measured specimens; SD – standard deviation, *pt* – ratio of the length of a given structure to the length of the buccal tube expressed as a percentage).

Character	N	Range						Mean		SD		Holotype	
		µm			*pt*			µm	*pt*	µm	*pt*	µm	*pt*
Body length	17	329	–	553	–	–	–	448	–	60	–	515	–
Peribuccal papillae length	12	6.8	–	10.4	*13.2*	–	*16.6*	9.0	*14.9*	1.1	*1.0*	9.3	*15.3*
Lateral papillae length	8	5.1	–	8.4	*10.0*	–	*13.0*	6.7	*11.3*	1.0	*0.9*	6.1	*10.0*
Buccal tube													
Length	17	44.8	–	65.6	–	–	–	58.4	–	6.5	–	60.9	–
Stylet support insertion point	15	31.2	–	45.8	*69.3*	–	*73.2*	40.8	*70.6*	4.7	*1.1*	43.9	*72.1*
Anterior width	16	14.0	–	23.0	*29.5*	–	*37.9*	19.0	*32.7*	2.5	*2.4*	20.1	*33.0*
Standard width	14	13.0	–	20.7	*27.5*	–	*36.4*	17.7	*31.0*	2.3	*2.6*	19.8	*32.5*
Posterior width	14	12.7	–	20.1	*26.6*	–	*33.5*	16.9	*29.6*	2.2	*2.3*	18.9	*31.0*
Standard width/length ratio	14	28%	–	36%	–	–	–	31%	–	3%	–	33%	–
Posterior/anterior width ratio	14	88%	–	97%	–	–	–	91%	–	3%	–	94%	–
Claw 1 lengths													
External primary branch	16	11.0	–	15.2	*19.5*	–	*24.6*	13.0	*21.9*	1.2	*1.6*	13.8	*22.7*
External base + secondary branch	15	9.6	–	14.9	*19.5*	–	*23.4*	12.6	*21.2*	1.4	*1.0*	13.8	*22.7*
External spur	7	2.8	–	3.7	*4.7*	–	*5.6*	3.2	*5.2*	0.3	*0.3*	?	?
External branches length ratio	14	87%	–	103%	–	–	–	97%	–	5%	–	100%	–
Internal primary branch	16	10.9	–	14.0	*19.4*	–	*24.6*	12.4	*20.9*	0.9	*1.4*	12.7	*20.9*
Internal base + secondary branch	16	9.0	–	14.0	*18.9*	–	*21.7*	12.1	*20.4*	1.4	*0.8*	12.8	*21.0*
Internal spur	13	2.8	–	3.6	*4.7*	–	*6.5*	3.1	*5.2*	0.3	*0.5*	3.2	*5.3*
Internal branches length ratio	15	83%	–	103%	–	–	–	98%	–	7%	–	101%	–
Claw 2 lengths													
External primary branch	15	10.6	–	15.3	*20.5*	–	*26.0*	13.3	*22.4*	1.2	*1.5*	14.6	*24.0*
External base + secondary branch	14	9.3	–	13.7	*18.8*	–	*21.5*	12.2	*20.5*	1.4	*0.7*	12.5	*20.5*
External spur	8	3.1	–	4.1	*4.9*	–	*6.7*	3.4	*5.5*	0.3	*0.6*	4.1	*6.7*
External branches length ratio	13	78%	–	103%	–	–	–	92%	–	7%	–	86%	–
Internal primary branch	14	10.9	–	15.0	*19.2*	–	*24.3*	12.5	*21.3*	1.1	*1.7*	13.5	*22.2*
Internal base + secondary branch	15	9.0	–	14.2	*18.0*	–	*22.3*	12.1	*20.2*	1.5	*1.0*	12.9	*21.2*
Internal spur	12	2.6	–	4.6	*4.3*	–	*6.9*	3.4	*5.7*	0.6	*0.7*	3.7	*6.1*
Internal branches length ratio	13	82%	–	103%	–	–	–	95%	–	8%	–	96%	–
Claw 3 lengths													
External primary branch	17	10.8	–	15.2	*19.0*	–	*26.5*	13.2	*22.6*	1.4	*1.7*	?	?
External base + secondary branch	16	9.5	–	15.7	*19.1*	–	*24.7*	12.0	*20.7*	1.6	*1.4*	?	?
External spur	7	3.0	–	4.0	*4.9*	–	*6.8*	3.3	*5.5*	0.4	*0.7*	?	?
External branches length ratio	16	79%	–	103%	–	–	–	92%	–	6%	–	?	–
Internal primary branch	17	10.7	–	14.1	*19.2*	–	*25.0*	12.4	*21.3*	1.1	*1.7*	?	?
Internal base + secondary branch	16	9.0	–	14.1	*17.8*	–	*21.8*	11.5	*19.7*	1.5	*1.1*	?	?
Internal spur	10	2.4	–	4.0	*4.1*	–	*6.8*	3.3	*5.7*	0.5	*0.9*	?	?
Internal branches length ratio	16	80%	–	102%	–	–	–	93%	–	7%	–	?	–
Claw 4 lengths													
Anterior primary branch	12	12.6	–	18.4	*23.7*	–	*28.9*	15.2	*25.8*	1.6	*1.9*	15.8	*25.9*
Anterior base + secondary branch	12	11.2	–	17.4	*22.6*	–	*27.5*	14.7	*25.0*	1.8	*1.6*	16.5	*27.1*
Anterior spur	7	2.7	–	5.2	*5.6*	–	*8.2*	3.7	*6.3*	0.8	*0.9*	4.2	*6.9*
Anterior branches length ratio	11	85%	–	104%	–	–	–	97%	–	6%	–	104%	–
Posterior primary branch	12	11.7	–	20.0	*23.7*	–	*31.4*	16.0	*27.3*	2.2	*2.0*	17.5	*28.7*
Posterior base + secondary branch	11	12.1	–	18.5	*24.0*	–	*28.9*	15.6	*26.5*	2.2	*1.8*	17.5	*28.7*
Posterior spur	7	2.9	–	5.2	*5.3*	–	*8.2*	3.8	*6.7*	0.9	*1.0*	4.4	*7.2*
Posterior branches length ratio	10	92%	–	103%	–	–	–	98%	–	4%	–	100%	–

Claws of the *Milnesium* type, stout (Figs 8–11). Primary branches on all legs with small, but distinct accessory points detaching from the branch at its greatest curvature (Fig. 10, empty arrowhead). Secondary branches of claws similar in length to primary branches and sometimes even longer. Secondary branches with rounded basal thickenings (Figs 9–11). Secondary branches on legs I–III with three points, secondary claws of anterior and posterior claws IV with four points (claw configuration: [3-3]–[4-4]). The fourth point on secondary branches is always very small and located near the base of the claw (Figs 10–11, arrowheads). Single, long transverse, cuticular bars present under claws I–III (Fig. 9, arrow).

***Males*** unknown.

***Eggs*** oval, smooth and deposited in the exuvium as in all other known *Milnesium* species.

###### DNA sequences.

We obtained good quality sequences for the applied molecular markers: 28S rRNA sequence (GenBank: MN191504), 638 bp long; COI sequence (GenBank: MN187057), 638 bp long; ITS-2 sequence (GenBank: MN239907), 392 bp long.

###### Remarks.

The fourth points on secondary branches of posterior claws can be barely visible or not visible at all in some positions of the specimens.

###### Type locality.

Madagascar, 22°37'04.5"S, 46°43'14.1"E, ca. 1198 m asl, Fianarantsoa Province, Ivohibory forest.

###### Etymology.

This species is named after Patricia Chapple Wright, an American primatologist and conservationist, best known for her studies on lemurs. She contributed to the establishment of the Ranomafana National Park in Madagascar. She also organized and led the expedition to the Ivohibory forest, during which several new species of tardigrades were found, including this species.

###### Type depositories.

The holotype and 23 paratypes (slides: MAD109/1, MAD109/3, MAD109/4, MAD109/5, MAD109/7) are deposited at the Department of Animal Taxonomy and Ecology, Adam Mickiewicz University in Poznań, Uniwersytetu Poznańskiego 6, Poznań, Poland, five paratypes (slides: MAD109/2) are deposited at the Institute of Zoology and Biomedical Research, Jagiellonian University, Gronostajowa 9,30-387, Kraków, Poland.

###### Morphological differential diagnosis.

The new species, by the presence of four points on secondary branches of claws IV, is most similar to *Mil. quadrifidum* Nederström, 1919, which is the only valid *Milnesium* species with four points on secondary branches of all claws. However, *Mil. wrightae* sp. nov. differs from *Mil. quadrifidum* not only by claw configuration ([4-4]–[4-4] in *Mil. quadrifidum* vs. [3-3]–[4-4] in *Mil. wrightae* sp. nov.), but also by the position of fourth points on secondary branches of claws IV (located near the base of the claw in the new species *vs.* near the top of the claw in *Mil. quadrifidum*). Additionally, all secondary branch points have similar length in *Mil. quadrifidum*, whereas the fourth points are very clearly smaller than the others in *Mil. wrightae* sp. nov.

###### Genotypic differential diagnosis.

The ranges of uncorrected genetic p-distances between the *Mil. wrightae* sp. nov. and species of the genus *Milnesium*, for which molecular marker sequences are available from GenBank (see Table 6 for details), are as follows:

1. 28S rRNA: 5.7–8.0% (6.7% on average), with the most similar being *Milnesium* sp. from North America (JX888585.1, JX888586.1, JX888587.1) (unpublished) and the least similar being *Mil. t. tardigradum* from Poland (KC138808.1 and KC138809.1) (unpublished);

2. COI: 17.7–38.4% (22.0 % on average), with the most similar being *Mil. variefidum* from UK (KT951663.1) (Morek et al. 2016) and the least similar being *Mil. t. tardigradum* from Spain (FJ435810.1) (Guil and Giribet 2012);

3. ITS-2: 25.6–36.3% (31.5% on average), with the most similar being *Mil. matheusi* sp. nov. (present studies) and the least similar being Mil. cf. granulatum from USA (MK681879.1) (Jackson and Meyer 2019).

## Conclusions

*Milnesium matheusi* sp. nov. and *Mil. wrightae* sp. nov. are new for science taxa, based on morphological as well as molecular characteristics. Until now, five *Milnesium* taxa have been reported from the African region, including Madagascar (i.e. *Mil. dornensis*, *Mil. matheusi* sp. nov. *Mil. tardigradum* s.s., *Mil. tetralamellatum* and *Mil. wrightae* sp. nov.). The presence of *Mil. tardigradum* s.s. in Madagascar needs confirmation and currently this record should be considered dubious.

## Supplementary Material

XML Treatment for Milnesium matheusi

XML Treatment for Milnesium wrightae
